# Development of automated machine learning models in predicting colorectal adenoma in metabolic-associated steatotic liver disease

**DOI:** 10.3389/fmed.2026.1852441

**Published:** 2026-05-28

**Authors:** Lu Liu, Huixian Zhang, Ziyu Zhao, Chunfang Xu, Shuting Qu

**Affiliations:** 1 Department of Gastroenterology, The Fourth Affiliated Hospital of Soochow University, Suzhou, China; 2 Department of Gastroenterology, The First Affiliated Hospital of Soochow University, Suzhou, China; 3 Department of Radiology, The First Affiliated Hospital of Soochow University, Suzhou, China

**Keywords:** clinical predictive models, colorectal adenoma, machine learning, non-alcoholic fatty liver disease, SHAP (Shapley Additive Explanations)

## Abstract

**Background:**

Metabolic-associated steatotic liver disease (MASLD) is recognized as the most common chronic liver disease worldwide. Identification of colorectal adenoma in MASLD is crucial to enable patients to take necessary preventive colonoscopy screening and treatment earlier. The main objective of this research is to build an automated machine learning (AutoML) model using a structured dataset to predict the presence or absence of colorectal adenoma in patients with MASLD.

**Methods:**

A study was conducted on a total of 506 patients with ultrasound-confirmed MASLD, and the data were used for training and validation. From the 29 selected parameters, we evaluated a prediction model for the prediction of colorectal adenoma in patients with MASLD, which was developed using univariate and multivariate logistic regression analyses. In addition, four AutoML-based predictive models were investigated and validated using the H2O machine learning platform.

**Results:**

The gradient boosting machine (GBM) model revealed an accuracy of 0.735, positive predictive value (PPV) of 0.729, negative predictive value (NPV) of 0.741, sensitivity of 0.754, specificity of 0.714, and area under the curve (AUC) of 0.791, performing significantly better than the other three AutoML models. The GBM model identified age, red blood cell (RBC) count, platelet (PLT) count, albumin, and alanine aminotransferase (ALT)-to-aspartate transaminase (AST) ratio as the most valuable predictors of colorectal adenoma in MASLD patients.

**Conclusion:**

The GBM model demonstrated good predictive ability for colorectal adenoma occurrence in MASLD patients.

## 1 Introduction

Metabolic-associated steatotic liver disease (MASLD) is the most common liver disease in industrialized countries, characterized by the accumulation of fat in more than 5% of hepatocytes without significant alcohol consumption (1). MASLD is an all-encompassing term for diseases ranging from simple steatosis to non-alcoholic steatohepatitis (NASH), NASH-related liver fibrosis, cirrhosis, and hepatocellular carcinoma (2–5). MASLD has been generally recognized as the hepatic manifestation of metabolic syndrome (MetS), which also includes the presence of abdominal obesity, insulin resistance, elevated blood pressure, impaired fasting glucose, and dyslipidemia. In addition, MASLD has been found to be closely associated with several extrahepatic comorbidities, including colorectal adenoma, cardiovascular disease (CVD), type 2 diabetes mellitus (T2DM), chronic kidney disease (CKD), and neurological diseases. Thus, MASLD is considered a multisystem disease with extrahepatic complications (6–8).

Accumulating evidence has shown that patients with MASLD have a higher risk of metabolic abnormalities associated with the development of neoplasia; consequently, extrahepatic neoplasia malignancy is the second leading cause of death among patients with MASLD, after cardiovascular disease (8). Several studies have reported that colorectal adenoma is the most common form of extrahepatic neoplasia in MASLD patients. A South Korean study involving 26,540 asymptomatic individuals who underwent abdominal ultrasound and first-time colonoscopy on the same day found that patients with MASLD had a higher prevalence of colorectal neoplasia than those without MASLD. This association remained significant, with an odds ratio (OR) of 1.10 (95% confidence interval [CI]: 1.03–1.17) for any colorectal neoplasia after adjustment for age, sex, smoking status, alcohol consumption, first-degree family history, aspirin use, and metabolic factors (9). A meta-analysis of 26 studies revealed a pooled OR of 1.37 (95% CI: 1.29–1.46) for the risk of colorectal adenomas in patients with MASLD (10). In recent years, some studies have attempted to develop models to predict the incidence of MASLD (11); however, accurate models for predicting colorectal adenoma as an extrahepatic manifestation of MASLD remain limited and are not well established.

Machine learning (ML) utilizes artificial intelligence to construct predictive models as an alternative to conventional statistical models, having more efficiency in detecting non-intuitive patterns from variables in statistical methods (12, 13). Several studies have indicated that using ML techniques improves the prediction of MASLD and other medical conditions. Pei’s research assessed fatty liver disease in normally screened populations (14). Elizabeth M. Brunt’s team carried out an analysis of hepatocyte ballooning identification in MASLD by AI-based ML approaches (15). Monica A. Konerman’s group used two ML models that incorporated longitudinal data to predict disease progression in chronic hepatitis C (16). The automated ML refers to one of the automated algorithms of ML for data cleaning, characteristics collection, model construction, and parameter selection. Few studies analyze how the technology performs compared to AutoML when using structured data; in addition, methods have more accuracy in models and less human effort in practicing ML expertise (17). This retrospective study aimed to fit a series of AutoML models using the H2O AutoML platform that predict colorectal adenoma in patients with MASLD.

## 2 Methods

### 2.1 Patients and data collection

We performed a retrospective study (*n* = 506) in which cases were MASLD patients with colorectal adenoma and controls were MASLD patients without adenoma. This study included patients with MASLD who had been screened for colonoscopy and excluded those whose clinical data were incomplete. The study data were collected from the Fourth Affiliated Hospital of Soochow University and the First Affiliated Hospital of Soochow University between January 2022 and January 2026. The Ethics Committee of the First Affiliated Hospital of Soochow University approved this retrospective study (Approval No.: 2022-232) and waived the requirement for individual informed consent. All patients were informed that their anonymized clinical data might be used for research purposes.

**Figure 1:**
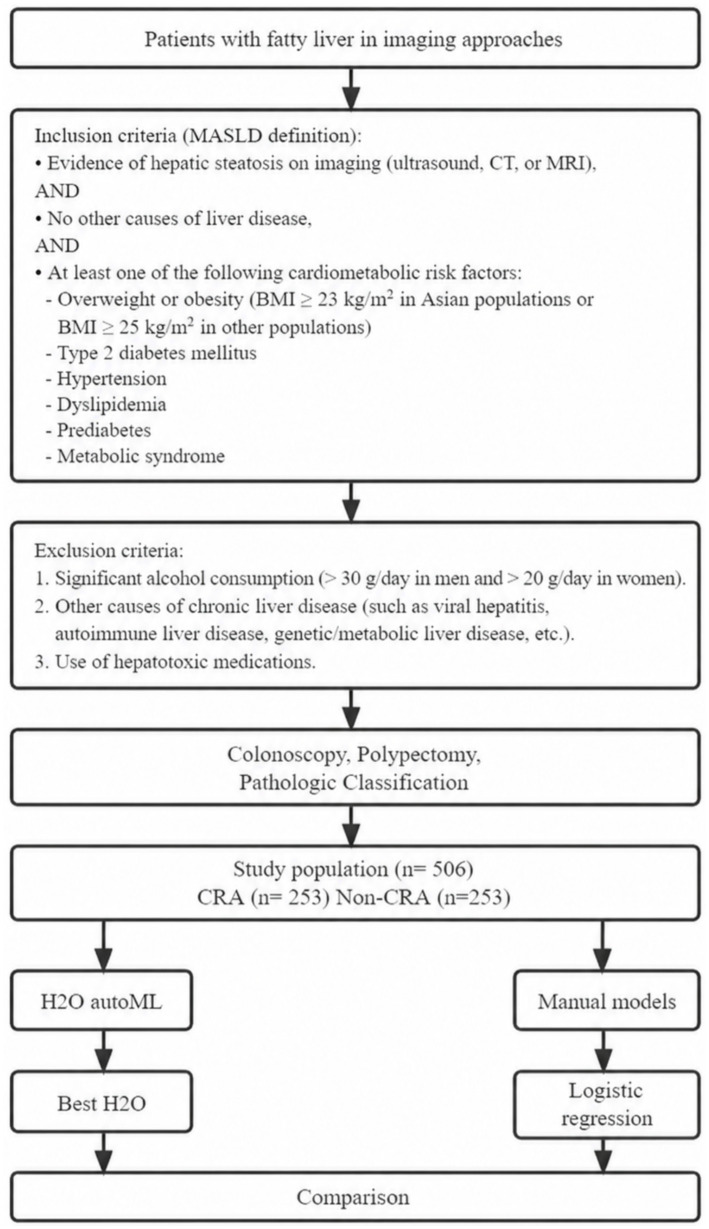
The flowchart in AutoML models.

All individuals were divided into two groups: colorectal adenoma and negative result in colonoscopy among MASLD patients. Patients with MASLD were diagnosed based on the presence of hepatic steatosis confirmed by imaging approaches and the absence of secondary causes for fatty liver diseases, especially chronic liver disease (such as viral hepatitis or autoimmune hepatitis) and alcohol consumption (>210 g/week in men and >140 g/week in women) (18). In addition, patients with or without colorectal adenoma were screened by colonoscopy and biopsied if any polypoid lesion was resected. Since the objective was to evaluate the presence of colorectal adenoma in MASLD, the analysis was restricted to patients who underwent complete colonoscopy with adequate bowel preparation and had no previous history of cancer or inflammatory bowel disease. Clinical details, including weight, height, body mass index (BMI), blood pressure, sex, and date of birth, were recorded. Blood tests were performed after fasting for 8 h. Liver, renal, and lipid biochemical parameters, including fasting serum glucose, albumin, total bilirubin (TBil), direct bilirubin (DBil), indirect bilirubin (IBil), gamma-glutamyltransferase (GGT), alanine aminotransferase (ALT), aspartate transaminase (AST), ALT-to-AST ratio, cholesterol, triglycerides, high-density lipoprotein (HDL), low-density lipoprotein (LDL), creatinine, urea, and uric acid (UA), were obtained. Hematological indicators, including white blood cells (WBCs), red blood cells (RBCs), platelets, hemoglobin, lymphocytes, monocytes, neutrophils, neutrophil-to-lymphocyte ratio (NLR), and lymphocyte-to-monocyte ratio (LMR), were measured. In total, 29 routine clinical and laboratory parameters were collected. The flowchart of the study is shown in Figure 1.

### 2.2 Logistic regression model building

The difference in the variables between patients with colorectal adenoma and those without colorectal adenoma was tested using logistic regression analyses. The logistic regression method was performed using the least absolute contraction selection operator (LASSO) regression model to identify the best predictive factors. The performance of the prediction model was assessed using the receiver operating characteristic (ROC) curve and the calibration curve. A nomogram was built based on the independent predictive factors identified from the multivariate analysis.

### 2.3 AutoML model construction and validation

The data were split into training (80%) and validation (20%) datasets randomly. Models were built using the H2O AutoML platform (version 3.36.0.2) as a scalable open-source platform for ML. Algorithms include a random grid of gradient boosting machines (GBMs), a fixed grid of generalized linear models (GLMs), an extremely random forest (XRF), and a random grid of deep learning (DLs) in a stacking ensemble. Default stopping strategies (number of models based = 30, the maximum runtime in seconds = 20) were performed on the training set. After training the base models, the models were ranked by a default metric, which used the cross-validated performance on training data. We reported the confusion matrix, consisting of accuracy, positive predictive value (PPV), negative predictive value (NPV), sensitivity, specificity, and AUCs for evaluating the prediction performance of models. To exhibit the visualized presentation of the association between features and outcomes, we implemented SHapley Additive Explanations (SHAP) and Local Interpretable Model-Agnostic Explanations (LIME, version 0.5.1). SHAP is an additive feature attribution method that gives an explanation of the overall performance of the contribution of a feature. The output of LIME is a series of explanations that indicate the contribution of key features to the predicted outcome in randomly selected individual patients. Additionally, a partial dependence plot (PDP) is also used to show the marginal effect of features on the predicted outcome.

### 2.4 Statistical analysis

Continuous data are presented as mean ± standard deviation (if normally distributed) or medians with interquartile ranges (if skewed). For normally distributed continuous variables, an unpaired *t*-test was used, whereas for non-parametric measures, the Mann–Whitney *U*-test was used. A two-sided *p*-value of < 0.05 was considered statistically significant. The statistical analyses were performed using R software (version 4.1.0).

### 2.5 Model comparison and assessment

We compared the performance of the logistic regression statistical model and four AutoML models to predict colorectal adenoma successfully. The prediction ability of the models was analyzed by the AUC. Additionally, in four AutoML models, prediction performance was assessed by accuracy, PPV, NPV, sensitivity, and specificity.

## 3 Results

### 3.1 Demographic and clinical characteristics of patients

A total of 506 MASLD patients who met the diagnostic criteria for MASLD, involving 253 colorectal adenoma patients and 253 MASLD patients without colorectal adenoma, were included in the study (Table 1). Descriptive and clinical characteristics of the study participants were classified by the presence or absence of colorectal adenoma. The 506 patients were randomly split into the training set and the validation set at a ratio of 8 to 2 (*n* = 405 in the training set and *n* = 101 in the validation set). The majority of the patients were male (68%) in the colorectal adenoma group, with a median age of 58 years. Patients with colorectal adenoma were typically male and older and had higher blood pressure levels and lower values for blood test indicators such as RBC count, platelet count, albumin, and LDL levels.

**Table 1:** Descriptive and clinical characteristics of the patients.

Variables	Sex	CRA (*n* = 253)	Non-CRA (*n* = 253)	*p*-value
Sex (%)	Male	172 (68%)	204 (80.6%)	0.002
	Female	81 (32%)	49 (19.4%)	
Age (year) (median [IQR])		58.00 [51.50, 64.50]	57.00 [50.00, 60.00]	0.033
BMI (kg/m^2^) (median [IQR])		26.34 [24.18, 27.66]	26.20 [24.57, 27.69]	0.593
SBP (mmHg) (median [IQR])		133.00 [122.50, 142.00]	131.00 [121.00, 140.00]	0.445
DBP (mmHg) (mean [SD])		82.63 (9.08)	82.91 (10.96)	0.753
WBC (×10^9^/L) (median [IQR])		6.14 [5.23, 7.20]	6.14 [5.21, 7.35]	0.983
RBC (×10^9^/L) (mean (SD))		4.71 (0.45)	4.99 (0.46)	<0.001
PLT (×10^9^/L) (median [IQR])		209.00 [172.50, 244.00]	221.00 [189.50, 260.50]	<0.001
L (*10^9^/L) (median [IQR])		1.95 [1.61, 2.35]	2.05 [1.70, 2.47]	0.087
M (*10^9^/L) (median [IQR])		0.42 [0.34, 0.54]	0.44 [0.36, 0.54]	0.257
N (*10^9^/L) (median [IQR])		3.46 [2.79, 4.29]	3.39 [2.71, 4.26]	0.285
NLR (median [IQR])		1.76 [1.40, 2.30]	1.69 [1.32, 2.12]	0.009
LMR (median [IQR])		4.72 [3.73, 5.62]	4.64 [3.76, 5.70]	0.533
GLU (mmol/L) (median [IQR])		5.32 [4.86, 5.90]	5.41 [5.06, 6.01]	0.117
Albumin (g/L) (median [IQR])		43.50 [39.85, 45.85]	45.30 [43.85, 46.90]	<0.001
TBil (μmol/L) (median [IQR])		14.70 [11.40, 17.70]	14.60 [11.70, 18.65]	0.982
DBil (μmol/L) (median [IQR])		4.40 [3.30, 5.50]	4.20 [3.30, 5.60]	0.188
IBil (μmol/L) (median [IQR])		10.30 [7.90, 12.40]	10.40 [8.15, 13.40]	0.324
AST (U/L) (median [IQR])		21.10 [17.85, 27.15]	23.20 [19.50, 28.45]	0.299
ALT (U/L) (median [IQR])		24.30 [17.50, 38.10]	24.80 [17.60, 33.90]	0.058
GGT (U/L) (median [IQR])		32.50 [21.65, 49.85]	35.6 [25.65, 54.15]	0.179
AST/ALT (mean [SD])		0.96 (0.66)	0.99 (0.34)	0.481
TCh (mmol/L) (median [IQR])		4.88 [4.25, 5.37]	5.13 [4.47, 5.76]	0.001
TG (mmol/L) (median [IQR])		1.82 [1.32, 2.42]	1.89 [1.40, 2.60]	0.762
HDL (mmol/L) (median [IQR])		1.06 [0.91, 1.23]	1.09 [0.94, 1.27]	0.457
LDL (mmol/L) (median [IQR])		2.90 [2.30, 3.41]	3.11 [2.53, 3.68]	0.001
Cr (μmol/L) (median [IQR])		66.8 [57.2, 77.9]	71.2 [62.20, 79.40]	0.002
Urea (mmol/L) (median [IQR])		5.40 [4.50, 6.10]	5.40 [4.70, 6.20]	0.21
UA (μmol/L) (median [IQR])		377.40 [320.30, 429.10]	408.00 [350.90, 474.85]	<0.001

BMI, body mass index; CRA, colorectal adenoma; SBP, systolic blood pressure; DBP, diastolic blood pressure; N, neutrophil; L, lymphocyte; M, monocytes; NLR, neutrophil-to-lymphocyte ratio; LMR, lymphocyte-to-monocyte ratio; AST, aspartate aminotransferase; ALT, alanine aminotransaminase; GLU, glucose; GGT, gamma-glutamyl transpeptidase; TCh, total cholesterol; TG, total triglycerides; TBil, total bilirubin; DBil, direct bilirubin; IBil, direct bilirubin; HDL, high-density lipoprotein; LDL, low-density lipoprotein; Cr, creatinine; UA, uric acid.

### 3.2 Univariate and multivariate logistic regression analysis

**Figure 2:**
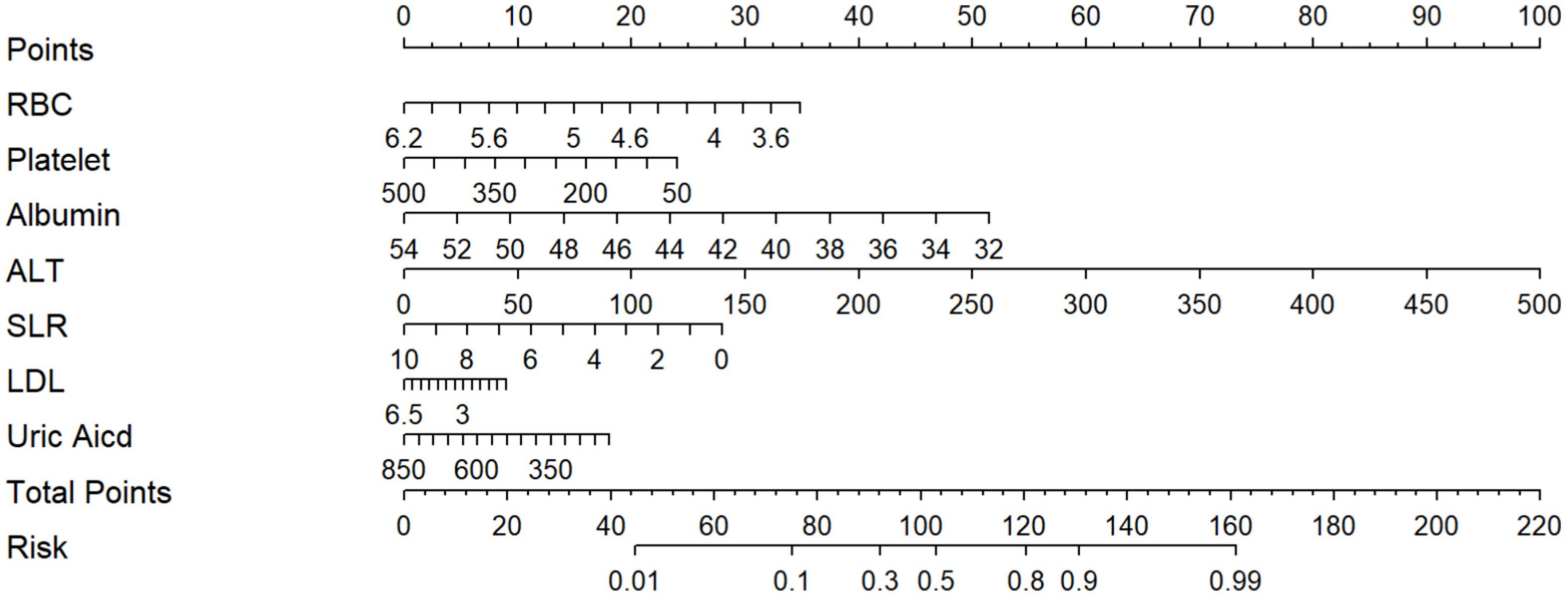
Nomogram of the logistic regression model.

**Figure 3:**
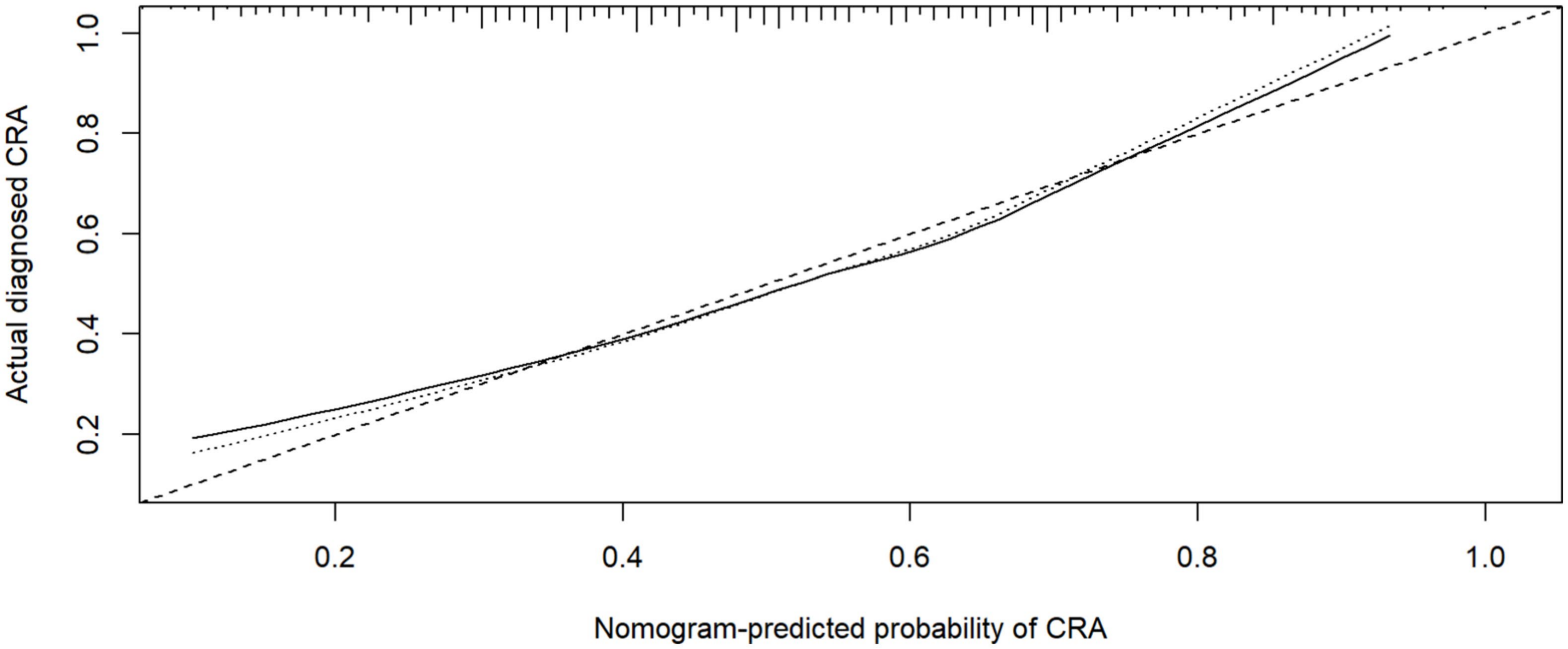
Calibration curves of the validation set in the logistic regression model.

Seven variables were selected in the univariate analysis and LASSO regression model, including RBC count, platelet count, albumin, ALT, AST-to-ALT ratio, LDL, and uric acid. Of the seven variables, ALT was presented as the risk factor, whereas the other six variables were presented as protective factors in developing colon adenoma in MASLD individuals. The nomogram was developed using the independent predictive factors (Figure 2). The calibration curves of the validation set are shown in Figure 3. The ROC curve of the validation set was presented, and its AUC was 0.636.

### 3.3 Automated machine learning analysis

**Figure 4:**
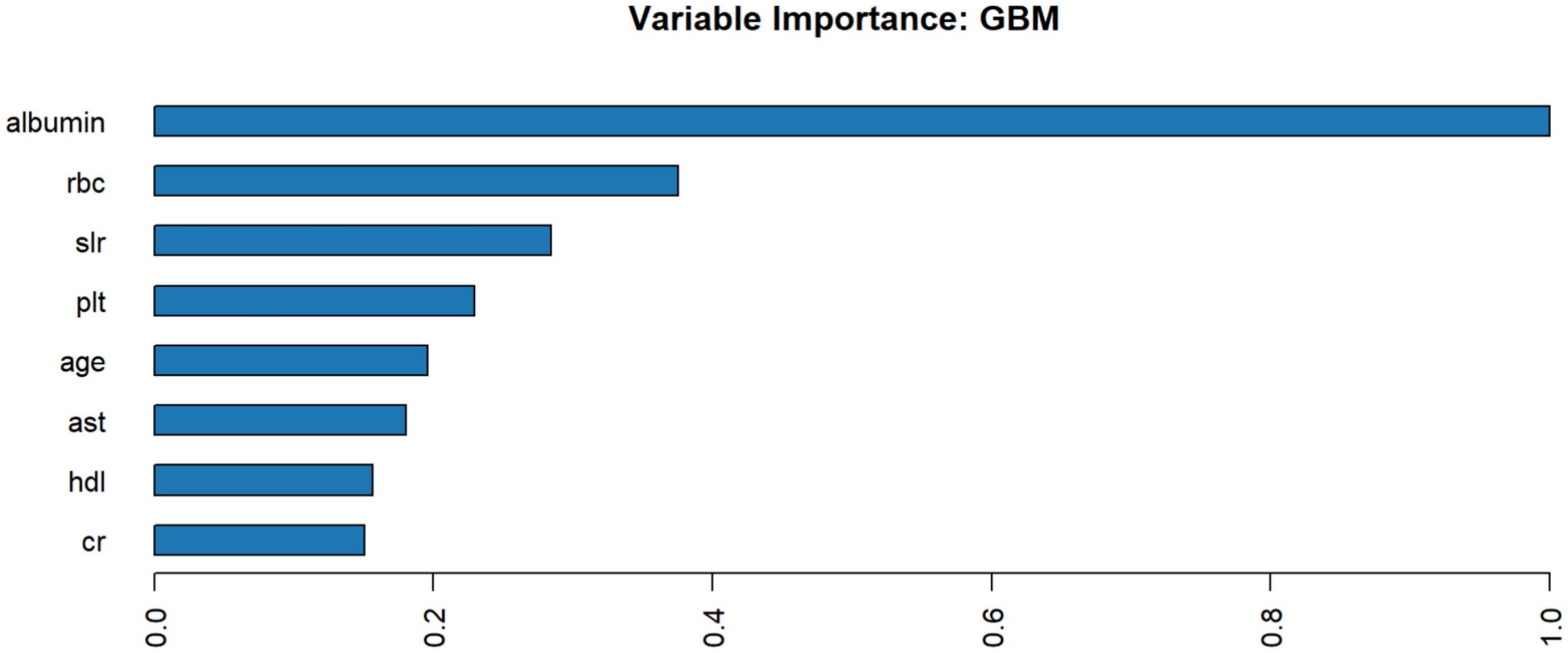
Importance of variables in the GBM model.

**Figure 5:**
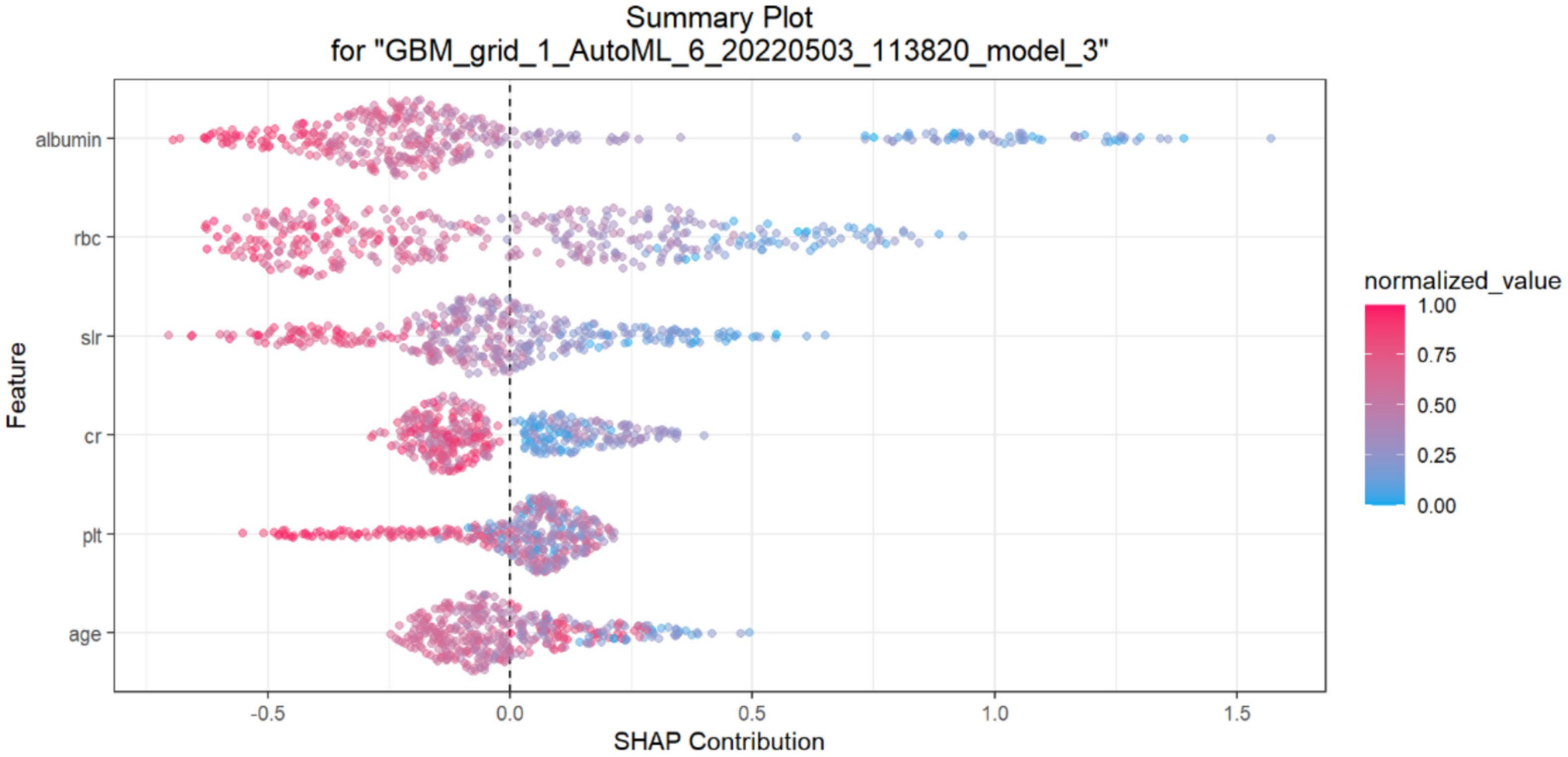
SHAP chart from the GBM model.

**Figure 6:**
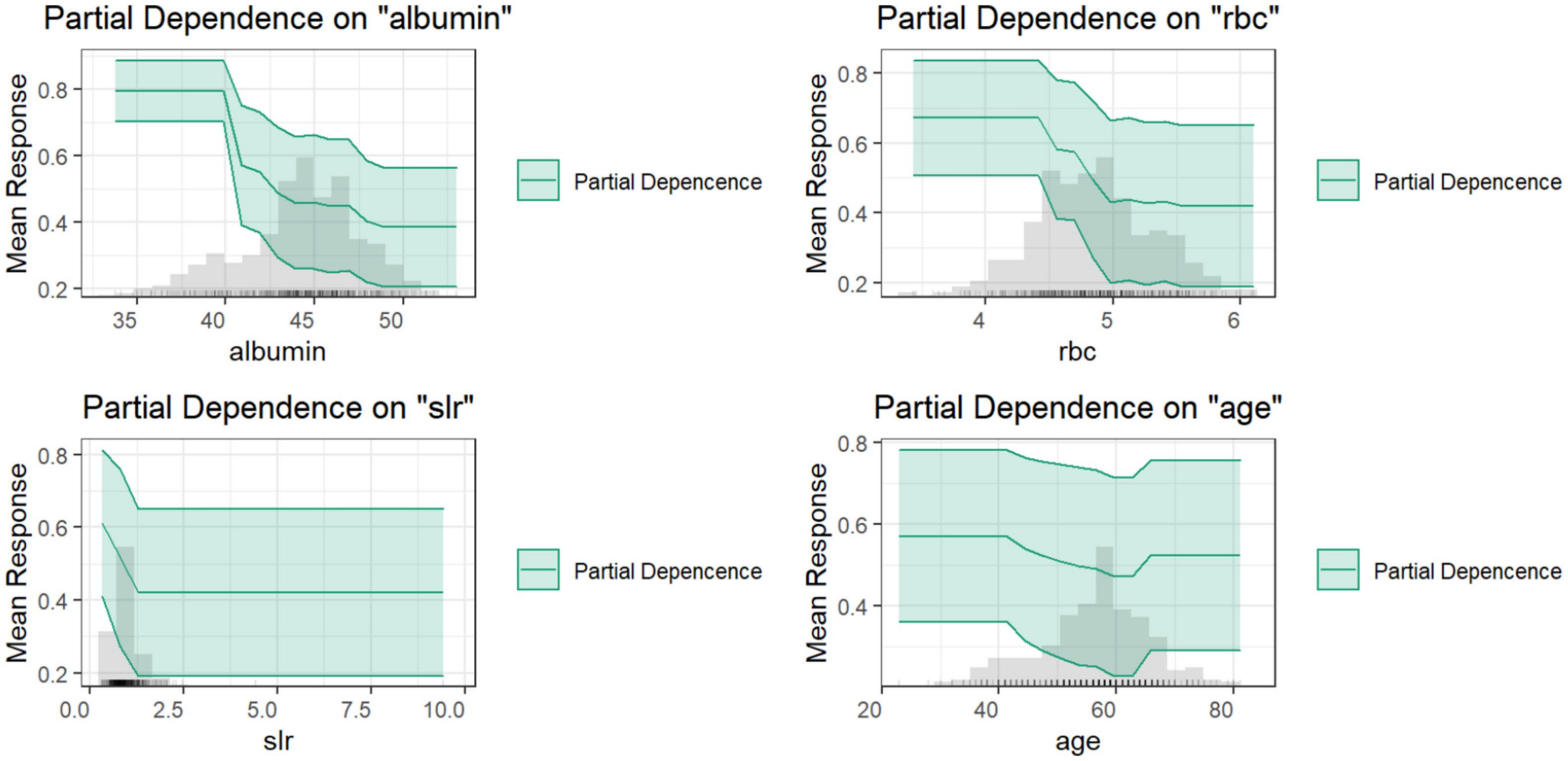
Partial dependence plots (PDPs) of four important variables from the GBM model. Slr.

**Figure 7:**
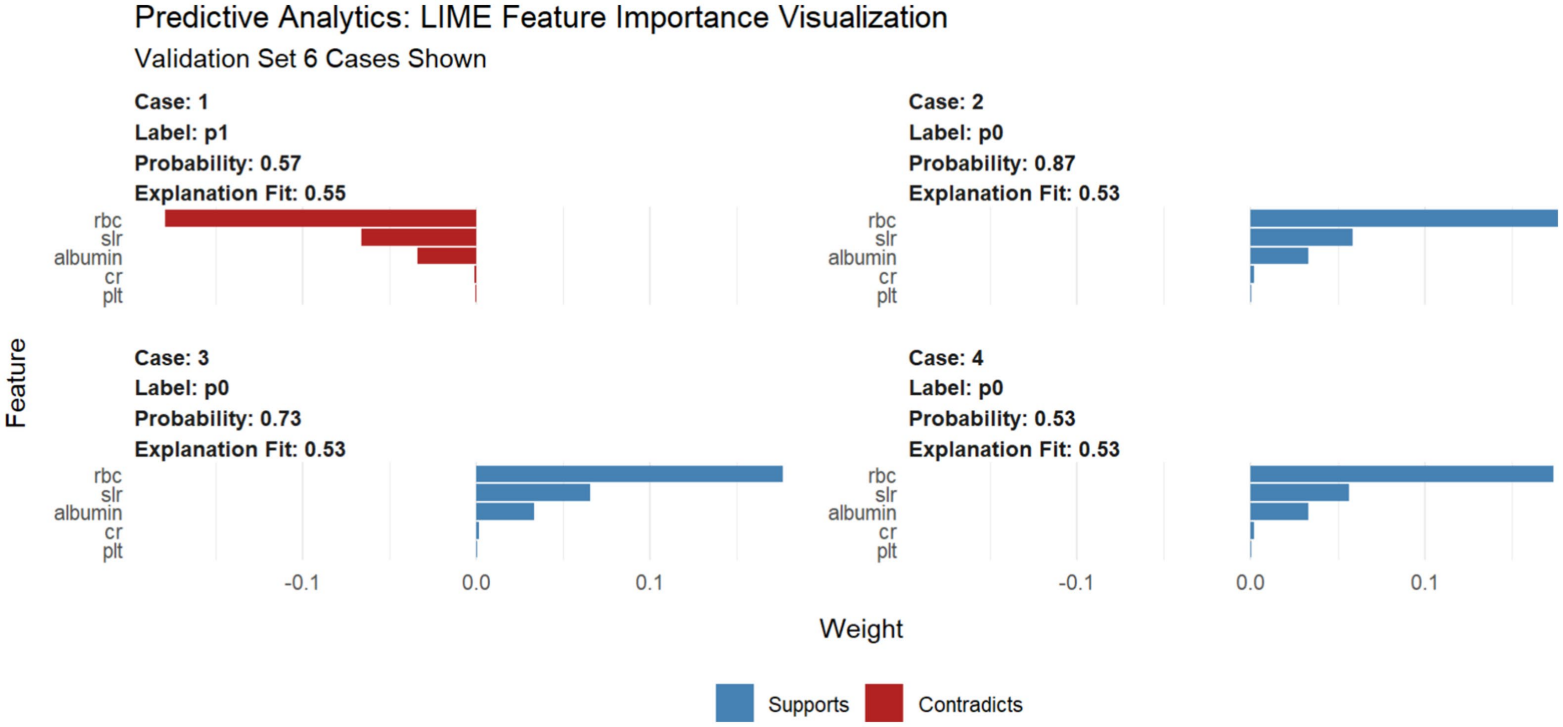
LIME from the GBM model.

A total of 29 models were developed based on four machine learning algorithms, including GBM, GLM, DL, and DRF, and poor interpretability stacked ensemble models were removed. Five-fold cross-validation was applied to all base models, with the same fold assignments maintained across algorithms. Feature selection using the LASSO regression model reduced the initial 29 variables to 7 inputs for all AutoML models to mitigate overfitting. In the colorectal adenoma group, the model with the highest value of AUC was GBM, indicating a comprehensive evaluation for the validation samples. The evaluation of the four AutoML models on the colorectal adenoma group data is shown in Table 2. The GBM algorithm achieved a higher value of AUC than the GLM, DL, and DRF algorithms (0.791, 0.726, 0.727, and 0.730, respectively). The accuracy was 0.735, 0.664, 0.628, and 0.584 in the GBM, GLM, DL, and DRF models, respectively, according to the confusion matrix of the four AutoML models. In the GBM model, as presented in Figure 4, albumin showed the leading feature of all variables, followed by RBCs, AST-to-ALT ratio, PLT, age, and other predictive factors. To gain insights into the impact of each predictor on the output of the AutoML model, we computed SHAP values for the GBM model (Figure 5). The horizontal axis represents the Shapley value, with values greater than 0 indicating a positive contribution to colorectal adenoma progression. The left longitudinal coordinate indicates the important order of features in reverse. The right longitudinal coordinate indicates the feature values from low to high. PDP is the application of the visualization of the linear relationship between the output and a variable. We examined the effects of albumin, RBCs, AST-to-ALT ratio, and PLT, which were the top four key variables in the GBM model, on the predicted output (Figure 6). From this figure, the relationship between the four features and the prediction of patient outcomes can be seen. The impact of albumin on the output increased when the value reduced from 48 to 40; RBCs’ impact fell when the value changed from 4.4 to 5.0; the AST-to-ALT ratio’s impact decreased when the value reached near 1.25; and the impact of PLT exhibited a complex trend, which slightly increased when the value changed from 150 to 170, remained the same impact, and then declined when the value exceeded 240. We next used LIME to exhibit how the key features contribute to the outcome impact at the individual level. Case 2 had a high probability of 0.87 for not developing colorectal adenoma, predicted by the GBM model due to the high values of the RBCs, AST-to-ALT ratio, and albumin (Figure 7).

**Table 2:** Results for various AutoML models in the validation set.

Model	Accuracy	Sensitivity	Specificity	PPV	NPV	AUROC
GBM	**0.735**	0.754	**0.714**	**0.729**	**0.741**	**0.791**
GLM	0.664	0.772	0.554	0.638	0.705	0.726
DL	0.628	0.807	0.446	0.597	0.694	0.727
DRF	0.584	0.550	0.846	0.965	0.196	0.730

AutoML, automated machine learning; GBM, gradient boosting machine; GLM, generalized linear model; DL, deep learning; DRF, random forest model; AUROC, area under the receiver operating characteristic (ROC) curve; PPV, positive predictive value; NPV, negative predictive value.

The highest values for each metric are marked in bold.

### 3.4 Comparisons between logistic regression and AutoML models

Based on the various models, we constructed and compared these models for predicting colorectal risk in patients with MASLD. Among these models, the GBM model achieved the highest AUC of 0.791, accuracy of 0.735, specificity of 0.714, PPV of 0.729, and NPV of 0.741. The other four are also good models for predicting the risk, with the AUC being relatively high at 0.636 for the logistic regression model, 0.726 for the GLM, 0.727 for the DL model, and 0.730 for the DRF model. The DL model had the highest value of sensitivity (0.807). The logistic regression model achieved an AUC of only 0.636, indicating limited discriminative ability. In contrast, all four AutoML models outperformed the logistic regression model, with GBM achieving the highest AUC (0.791).

## 4 Discussion

In the present study, we applied logistic regression and AutoML algorithms to create the predictive colorectal adenoma risk tool for MASLD patients. The GBM from the AutoML models reached the highest AUC value of above 0.79, with the accuracy, sensitivity, and specificity of above 0.70. The five most important features for developing colorectal adenomas from GBM were albumin, RBCs, AST-to-ALT ratio, PLT, and age. ALT also showed an important role in the disease progression in the logistic regression model.

Over the last decade, there is now growing evidence that MASLD can raise liver-related morbidity and mortality and also contribute to the clinical burden of several extrahepatic systems and regulatory pathways (19). A cohort of 12,853 South Korean individuals examined the risk of incidence of T2DM, which evaluated three risk factors, including obesity, insulin resistance, and fatty liver (defined by standard ultrasound) at a 5-year follow-up. After fully adjusting for potential confounders in models, the cohort data revealed that each of the three risk factors was independently associated with the incidence of T2DM. In particular, patients with fatty liver had an approximate doubling of the T2DM risk compared with individuals without fatty liver. There was a 14-fold increase in the risk of incidence of T2DM when the patient had three of these risk factors (20). Several cross-sectional studies have revealed that the prevalence of CVD is increased in patients with MASLD. A national-based cohort study in the United States has reported that the clinical CVD disease increased among the patients with MASLD (21). A retrospective cohort study of 5,517 women in South Korea has expressed that the association between MASLD and the risk of colorectal neoplasms had a significant relationship, in which the adjusted relative risk (95% CI) for colorectal cancer in MASLD patients was 3.08 (95% CI 1.02–9.34) (22). Colorectal adenoma is a widespread precancerous lesion in the intestinal tract, which is associated with extrahepatic neoplasia in MASLD (23). In a large outpatient cohort analysis from New York, the prevalence of colorectal adenoma was significantly higher in the group with MASLD than in healthy controls (OR = 2.09, 95% CI = 1.31–3.33, *p* = 0.002) (24).

In the study, we built the models for predicting colorectal adenomas in MASLD patients. To achieve a high prediction performance, four types of AutoML algorithms, including GBM, GLM, DRF, and DL, were used. Among the models, the DL model ranked first in sensitivity, and the GBM model reached the highest in the AUC, accuracy, and specificity. Both the logistic regression model and AutoML models demonstrated that albumin, age, RBCs, and PLT were the vital factors in the occurrence of colorectal adenoma in patients with MASLD.

We computed the SHAP to gain insights into the impact of the important predictor of the model. The SHAP analysis showed that albumin was the most important feature of the model, which was also in the first rank in the variable importance of the GBM model. As widely reported in the literature, albumin plays a key role in the nutritional and inflammatory status of the patients. In Antonella’s research, the level of serum albumin was lower in colorectal neoplasia patients compared with healthy controls, which indicated that patients with colorectal neoplasia had a higher risk of nutritional imbalance (25). As an acute-phase protein, a low concentration of albumin reflects a negative impact on prognosis. Steven Kazmierczak’s team has concluded that serum albumin might be used as a diagnostic and monitoring tool for chronic diseases such as gastrointestinal carcinoma, with the sensitivity and specificity of this diagnostic test being 87.4 and 85.7%, respectively (26). Previous research conclusions are consistent with our data. Age and sex are also reportedly widely recognized as important factors in the occurrence of colorectal adenoma in fatty liver patients. An analysis from Korea has figured out that the median age of the adenoma group was 2 years older than the non-adenoma group, and male individuals were five-fold more than female individuals. Another Korean team’s finding was also consistent with this result, which revealed that individuals in the colorectal adenoma group were significantly older and more male compared to the control group (27, 28). RBCs are a blood routine parameter for predicting the prognosis of colorectal cancer or adenoma, especially at the early stage. Peng’s study has shown that RBC count was significantly associated with a reduced risk of developing colorectal neoplasia (29). The major effect of PLT is known as hemostasis and thrombosis. In addition, PLT also participates actively in the progression of tumor growth. Some studies have shown that platelets and platelet-derived microparticles apply antiproliferative and cytotoxic effects on tumor cells in a cadherin-6-dependent manner. This might explain why the level of PLT in the adenoma group was lower than in the other one. However, the role in tumor growth remains controversial. Some literature has mentioned that PLT might promote the metastasis of tumor stage through epithelial-to-mesenchymal transition and endothelial activation (30–32). ALT was most highly correlated with fat accumulation in hepatocytes, which was also found to be associated with colorectal adenoma in the research (33). The AST-to-ALT ratio is considered a parameter of hepatitis. Several researchers used the ratio to evaluate MASLD or NASH. It reflected the hepatic pattern of hepatocyte damage or altered insulin sensitivity (34, 35).

## 5 Conclusion

In this study, we built five predictive models using traditional logistic regression and AutoML models. Using the structured data, we constructed a series of models for predicting colorectal adenoma in MASLD patients. Compared with the traditional logistic regression analysis, AutoML demonstrated higher accuracy and efficiency with less human effort. AutoML operates using various machine learning algorithms and multiple classifiers to predict the outcomes. In addition, AutoML provides a visualized explanation of the model at the feature and individual levels (36). The discrepancy between the logistic regression model and the GBM model regarding ALT and the AST-to-ALT ratio reflects fundamental differences between linear and tree-based models. In the presence of collinearity, the logistic regression model may assign unstable coefficients, whereas the GBM model preferentially selects the more informative derived feature (AST-to-ALT ratio), which integrates both transaminases. This ratio is clinically significant as it reflects hepatic inflammation and may be associated with insulin resistance. Several limitations in our study should be noted. First, our research is a retrospective study, which might affect the accuracy of the models in a prospective clinical study. Moreover, models in the study were developed and validated on the basis of constructive data obtained from a single center. Inclusion of a test set from another medical center may further improve the performance of the models.

## Data Availability

The original contributions presented in the study are included in the article/supplementary material, further inquiries can be directed to the corresponding author.
